# Unsafe and unequal: a decomposition analysis of income inequalities in fear of crime in northern Sweden

**DOI:** 10.1186/s12939-018-0823-z

**Published:** 2018-08-01

**Authors:** Beáta Vivien Boldis, Miguel San Sebastián, Per E. Gustafsson

**Affiliations:** Epidemiology and Global Health, Department of Public Health and Clinical Medicine, Umeå University, 901 87 Umeå, Sweden

**Keywords:** Decomposition analysis, Concentration index, Income inequality, Fear of crime, Gender, Sweden

## Abstract

**Background:**

Fear of crime is not solely an individual concern, but as a social determinant of health structured by gender it also poses a threat to public health. Social inequalities are thought to represent a breeding ground for fear of crime, which subsequently may contribute to social inequalities in health. However, little research has focused on social inequalities in fear of crime, particularly in Sweden where the level of fear of crime and income and gender inequalities are comparatively low. With a conceptual model as a point of departure, the present study aimed to estimate and decompose income-related inequalities and explore gender differences in fear of crime in northern Sweden.

**Methods:**

Participants (*N* = 22,140; 10,220 men and 11,920 women aged 16 to 84 years) came from the Health on Equal Terms cross-sectional survey with linked register data, carried out in the four northernmost counties of Sweden in 2014. Disposable income was used as the socio-economic indicator, fear of crime as the binary outcome variable, and sociodemographic characteristics, residential context, socio-economic and material conditions and psychosocial conditions as explanatory factors. Concentration curve and concentration index were used to estimate the income inequality in fear of crime, and decomposition analysis to identify the key determinants of the inequalities, in collapsed and gender-stratified analyses.

**Results:**

Substantial gender differences were found in the prevalence of fear of crime (20.8% in women and 3.5% and men) and among the contributing factors to fear of crime. Additionally, the analyses revealed considerable income inequalities in fear of crime in the northern Swedish context (C = − 0.219). Gender, socio-economic and material, and psychosocial conditions explained the most in income inequalities of fear of crime in the total population.

**Conclusions:**

The existing gender and socio-economic inequities need to be approached as a greater structural problem to mitigate inequalities in fear of crime. Further research is needed to reveal more aspects of income inequalities in fear of crime and to develop efforts to create safe environments for all.

## Background

Fear of crime is an emotional reaction towards the individual risk of criminal victimization that leads to mental and physical poor health [1, 2]. Moreover, its socio-economic and gendered unequal distribution makes it a possible social determinant not only of health but of inequalities in health. Previous research has mostly investigated fear of crime from a socio-ecological perspective [3–5], and to the authors’ knowledge there is no literature on the underpinnings of socio-economic inequalities in fear of crime. The present study seeks to contribute to filling this gap in the literature using as the point of departure northern Sweden, a comparatively socially equal and secure setting.

Fear of crime is increasing in the Swedish context despite being lower than in other non-Nordic European countries [6]. For example, according to the recent 2016 Swedish Crime Survey [7], as many as 19% of the respondents felt unsafe outdoors late at night, with the youngest and oldest women being the most fearful, and the proportion of respondents concerned that fear of crime affects their quality of life almost doubling since 2015 [7]. At the same time, the increasing income inequalities in Sweden [8] also raises worries about exacerbated inequalities in fear of crime. These observations imply that inequalities in fear of crime may be an important albeit understudied public health issue, particularly with regard to the underpinnings of such inequalities, which have not been comprehensively investigated nor explicitly conceptualized from a public health perspective. This leaves little guidance for policymakers to work towards an equal and safe life for all.

**Fig. 1:**
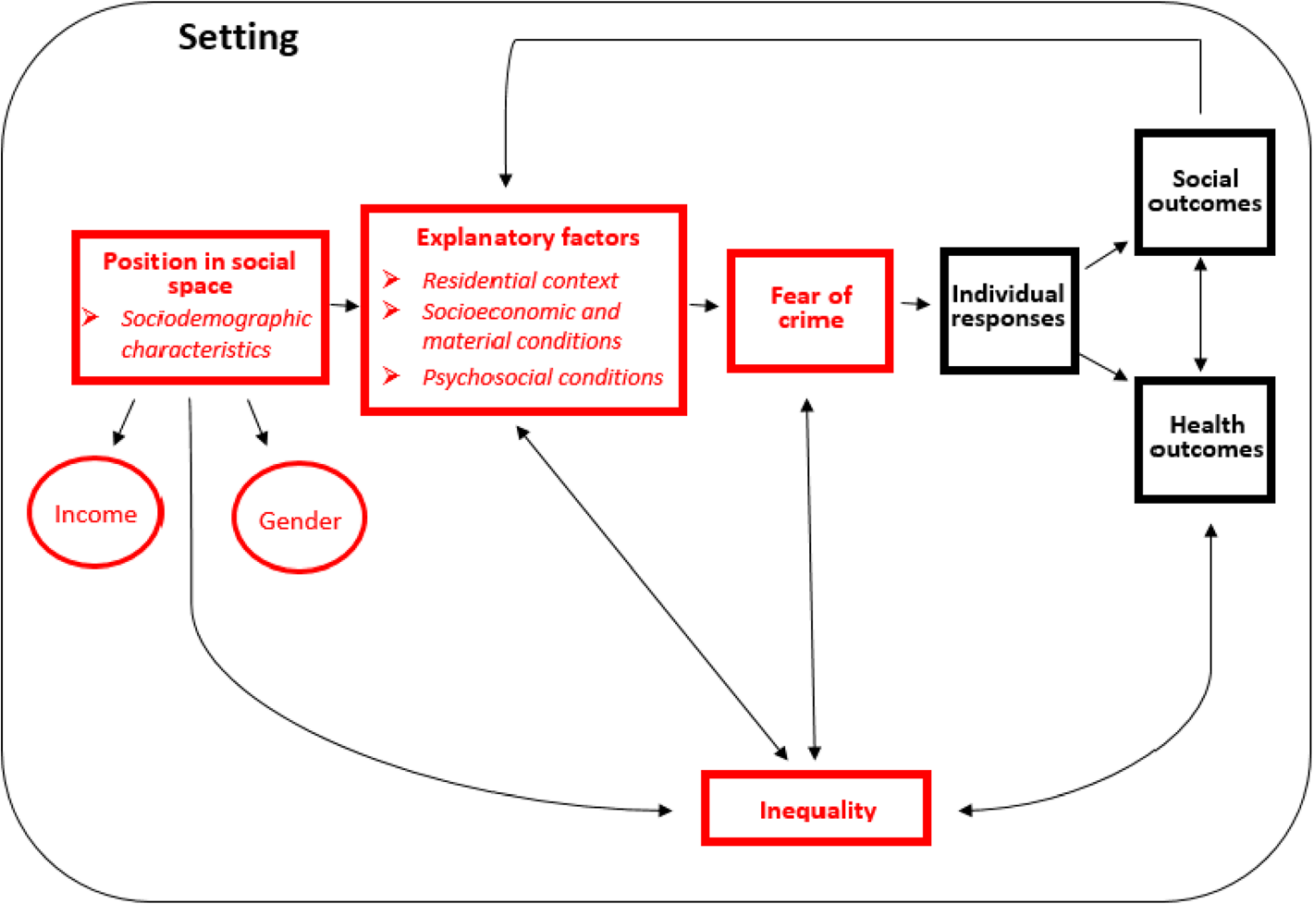
Conceptual framework of the role of fear of crime from a public health perspective, modified from Garofalo 1981. Areas of specific interest for the present study are indicated in red

In 1981, Garofalo described a conceptual framework called ‘A general model of the fear of crime and its consequences’ [1], a revised version of which is presented in Fig. 1, modified to more clearly frame the role of fear of crime from a public health perspective.

In the model, *position in social space* includes, for example, socio-economic status, gender, age, country of birth or ethnicity, and sexual orientation. By being the basis of social inequalities, social position has an ubiquitous influence on the other elements in the model, including experiences of and vulnerability to fear of crime [1, 9]. Two components of particular interest to the present study are *income* and *gender that determine the individual position in social space by operating within a socio-economical structure*.

According to Wilkinson and Pickett, income inequality contributes to violence and crime, which causes increased fear of crime in all layers of society [3]. An alternative perspective is offered by Hummelsheim et al. [4], who instead argue that crime rate has only a minor impact on fear of crime, while income inequality seems to be positively linked with fear of crime independently of actual crime levels [4, 5]. Thus, individuals who are worseoff might experience more fear, which would imply income inequalities in fear. Considering that income inequalities are increasing across the European region, including Sweden [8], income inequalities in fear of crime represent a particular cause of concern.

Fear of crime is, like economic inequalities, also intricately tied to gender, where women tend to report more fear of crime than men – even though men are victimized to a greater proportion than are women [10]. This apparent paradox further demonstrates fear of crime as a phenomenon at least partly independent of and distinct from actual crime levels and risks, and can from a feminist perspective be explained by the oppressed position occupied by women in the gender structure maintained in patriarchal societies, marked by a male dominance [11]. The deeply entrenched perceptions related to hegemonic ideologies about masculinity and femininity prescribe that women are more likely to be victimized because of their perceived vulnerability [12] and the apparent paradox can thus be traced to the inequitable gender structure rather than to women’s actual vulnerability.

*Explanatory factors* in Fig. 1 refer to conditions or experiences that could explain inequalities in fear of crime: those that might contribute to fear of crime and also are potentially unequally distributed by income. *Socio-economic and material conditions* are fundamental to the investigation of income inequalities in fear of crime. Fear of crime is principally incited by social inequities and economic disadvantages [13, 14], and individuals can only respond adequately to fear if they have appropriate socio-economic resource; − for example, buying a car instead of using a potentially unsafe public transportation [1]. Therefore, inequalities, such as income inequality, affect the quality and intensity of the response to fear by providing possibility to access different resources [1].

Furthermore, *psychosocial conditions* play a major role in the presence of inequality in fear of crime; for example, social participation and social capital can decrease the level of fear of crime [15]. Lastly, *residential context* seems to influence fear of crime [1, 9], for instance, urban areas where crime rates are higher, fear of crime is correspondingly greater than in rural and low crime rate areas [16–18].

According to Garofalo [1], individual responses to fear of crime can produce negative *social outcomes* – such as social distrust, alienation from social life and political distrust, throughout individual responses to fear of crime [19]. However, fear of crime is also influenced by the social outcomes themselves, since social outcomes can also play the role of explanatory factors, and a vicious circle or protective feedback may thus occur. Furthermore, individual responses to fear of crime lead to negative *health outcomes* [2, 20, 21], such as poor mental health and depression [21], and a decrease in physical activity to avoid fear and possible victimization [20, 21], which in turn may impact on social outcomes, and vice versa.

With the presented model as a conceptual point of departure, the present population-based study aimed 1) to estimate income inequalities in fear of crime, 2) to identify and measure key explanatory factors of income inequalities in fear of crime and, 3) to explore gender inequalities in fear of crime, income inequalities in fear of crime and determinants of income inequalities in fear of crime in northern Sweden.

## Methods

### Study population and data

Secondary data were obtained using the Norrland ‘Health on Equal Terms’ (HET) national cross-sectional survey from 2014, that has been carried out by the county councils in collaboration with the Public Health Agency of Sweden. Details about the survey procedures and questionnaire are found in technical reports [22, 23].The questionnaire was distributed in a collaboration between the Swedish National Public Health Agency, Statistics Sweden and the respective county councils of the four northernmost counties of Sweden: Västernorrland, Jämtland/Härjedalen, Västerbotten and Norrbotten.

All residents aged 16–84 years were identified as the target population. The sample frame comprised 704,099 individuals. Sampling was carried out with a two-steps probabilistic procedure. First, a random, national and -unstratified selection was performed from the national HET survey. Second, a regional random sample stratified by gender, age, county and municipality was conducted. The overall participation rate was approximately 50%, leading to a sample size of 25,667 individuals who answered either the postal or Web questionnaire. Further inclusion or exclusion criteria were not applied, and item non-response was handled by using complete case analysis, resulting in a total analytical number of 22,140 observations (approx. 43% of the invited and 86% of the respondents): 10,220 men and 11,920 women.

The HET questionnaire covers domains such as health, health behaviours, health-care use and psychosocial and material conditions. In addition, the survey data were, through the Swedish Personal Identity Number, linked to individual-level sociodemographic data such as annual income (from 2012), education level and country of birth, retrieved from the total population registers of Statistics Sweden.

### Measures

The fear of crime outcome variable was based on the question: ‘Do you ever avoid going out alone out of fear of being assaulted, robbed or otherwise victimized?’ and coded as no (0) or yes (1) (‘yes, sometimes’, ‘yes, often’). The answer options ‘yes, sometimes’ and ‘yes, often’ were collapsed, since the frequency of ‘yes, often’ was rather small; 1.5% in the total sample.

Annual disposable, individual income was obtained from the 2012 registers of Statistics Sweden, reflecting the remaining income after tax deductions and all positive and negative transfers. The mean individual income was 205,553 Swedish Krona (SEK) (29,768 US$, based on exchange rate from January 1, 2012) per year in the total sample, 232,602 SEK (33,685 US$) among men and 182,362 SEK (26,410 US$) among women.

Explanatory variables were chosen to capture the individual and social context of fear of crime, in accordance with the literature [6], and grouped together under four categories: sociodemographic characteristics; residential context; socio-economic and material conditions; and psychosocial conditions as presented in Fig. 1.

In the *sociodemographic characteristics category*, the following five variables were included:

- *Gender,* coded as man (1) and woman (2).
- *Age* was divided into four age ranges coded as 16–29 years (1); 30–44 years (2); 45–64 years (3) and 65–84 years (4).
- *Country of birth*, coded as Swedish (1) and non-Swedish (2).
- *Civil status,* coded as married or cohabitating (1), unmarried or not-cohabitating (2), divorced (3) and widower (4).
- *Sexual orientation,* coded as heterosexual (1) and LGBQ (Lesbian-Gay-Bisexual-Questioning) (2), consequently forming a binary variable of sexual orientation (see ref. [24] for more details).

*Residential context* covered the following two factors:

- *Municipality size*, coded as municipalities with population more than 50,000 (1), municipalities with population between 10,000 and 50,000 (2), municipalities with population less than 10,000 (3).
- *Residential ownership*, coded as resident-owned (1), rental (2) and other arrangements (live-in, student room, or other living arrangements) (3).

*Socio-economic and material conditions* covered the following five factors:

- Annual disposable *income* was also included as explanatory variable to avoid overestimation of other explanatory variables, which could correlate with income, as suggested by Wagstaff et al. [25]. It was divided into quintiles (5 coded as the highest income quintile and 1 as the lowest) [24, 26–29].
- *Education*, coded as high (three and more years of post-secondary education) (1), medium (3 years secondary education to 2 years post-secondary education) (2), and low (up to 2 years of secondary education) (3).
- *Labour market position*, coded as working (employed, self-employed, temporary leave of absence) (1), studying (2), retired (age retirement) (3) and non-employed (unemployed, long-term sick leave, early retirement due to ill-health, taking care of household and labour market programme) (4).
- *Low cash margin*, was based on whether the respondent would be able to get hold of 15,000 SEK in 1 week. Those who could get hold of 15,000 SEK were coded as ‘no’ (1), those who could not as ‘yes’ (2).
- *Difficulties to make ends meet*, whether the respondent had had difficulties in managing the regular expenses during the last 12 months coded as ‘no’ (1), and ‘yes’ (2).

In the *psychosocial conditions category*, the following four variables were included:

- *Social participation* was based on whether the respondent had taken part in activities during the last 12 months, such as ‘study circle/course at your workplace’, ‘study circle/course in your free time’, ‘trade union meeting’, ‘other association meeting’, ‘theatre/cinema’, ‘art exhibition’, ‘religious gathering’, ‘sporting event’, ‘written to the editor at newspapers/periodicals’, ‘demonstration of some kind’, ‘public entertainment e.g. night club, dance or similar’, ‘large family gathering’, ‘private party at someone’s home’, or ‘none of the above’. A positive response to one or more activity was coded as ‘yes’ (1), with no activity coded as ‘no’ (2).
- *Social trust* was based on whether the respondent can generally rely on other people coded as ‘yes’ (1) and ‘no’ (2).
- *Subjected to threat or violence* variable was based on the combination of two items: whether the respondent had been subjected to the threat or menace of violence and/or whether the respondent had been subjected to physical violence in the last 12 months, coded as ‘no’ (1) and ‘yes’ (2).
- *Degrading treatment* was based on whether the respondent had been treated in a way that was perceived as humiliating in the last 12 months, coded as ‘no’ (1) and ‘yes’ (2).

### Data analysis

**Fig. 2:**
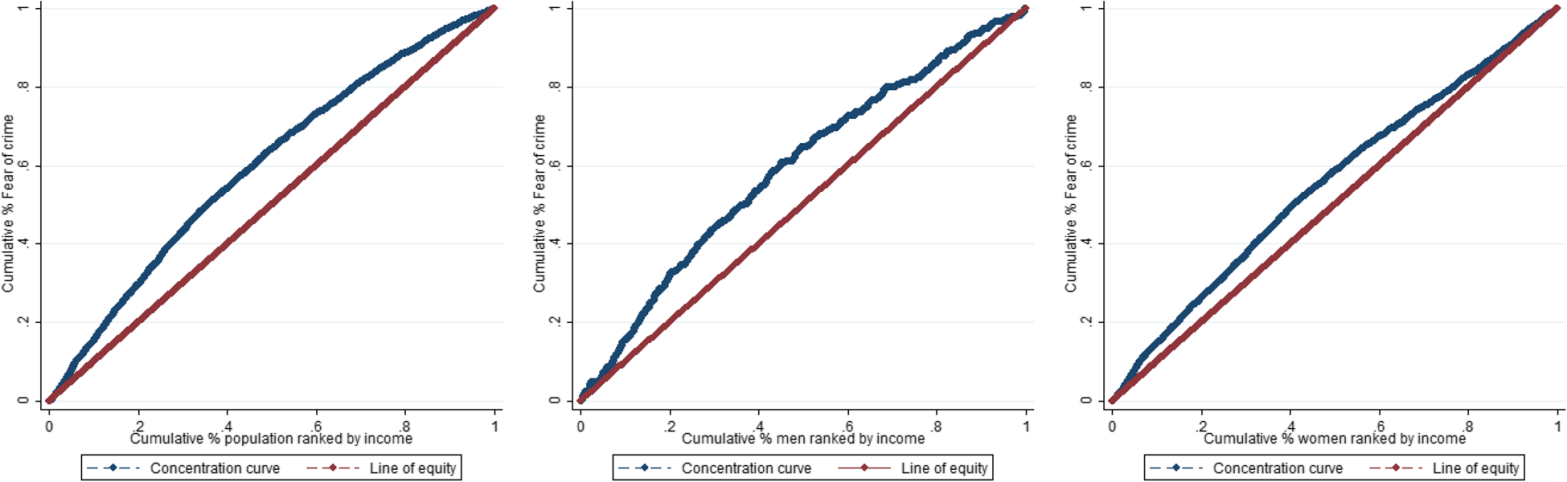
Concentration curves for cumulative proportion of fear of crime by ranked disposable income in the total sample, men and women, in 2014, northern Sweden

First, a descriptive analysis was run, on the total and gender stratified samples (Table 1). Then, to fulfil the first aim, concentration curves (CCs) and concentration indexes (Cs) were used for estimating the degree of income-related inequality in the distribution of the outcome variable fear of crime. The CC plots the cumulative percentage of the outcome (fear of crime) on the y axis, and the cumulative percentage of the sample ranked by the socio-economic indicator (individual disposable income) from the poorest to the richest on the x axis (Fig. 2) [30, 31]. A 45° diagonal line of equality indicates equal distribution of the outcome along the ranked indicator.

**Table 1:** Descriptive statistics of all variables in the total sample, women and men, in 2014, northern Sweden (*N* = 22,140)

	Frequencies (n, %) of participants reporting Fear of crime within each variable category											
	Total				Men				Women			
	No		Yes		No		Yes		No		Yes	
	n	%	n	%	n	%	n	%	n	%	n	%
	19,298	87.2	2842	12.8	9861	96.5	359	3.5	9437	79.2	2483	20.8
**Sociodemographic characteristics**												
*Gender*												
Men	9861	96.5	359	3.5	9861	96.5	359	3.5	–	–	–	–
Women	9437	79.2	2483	20.8	–	–	–	–	9437	79.2	2483	20.8
*Age*												
16–29 yrs	2720	80	680	20	1399	95.7	60	4.3	1381	69	620	31
30–44 yrs	4158	87.8	578	12.2	1982	97.1	60	2.9	2176	80.8	518	19.2
45–64 yrs	6134	90.1	674	9.9	3079	96.7	104	3.3	3055	84.3	570	15.7
65–84 yrs	6286	87.4	910	12.7	3461	96.3	135	3.8	2.825	78.5	775	21.5
*Country of birth*												
Swedish	18,263	87.3	2648	12.7	9416	96.7	326	3.4	8847	79.2	2322	20.8
Non-Swedish	1035	84.2	194	15.8	445	93.1	33	6.9	590	78.6	161	21.4
*Civil status*												
Married/Cohab	13,996	88.5	1827	11.6	7049	97.3	194	2.7	6947	81	1633	19
Unmarried/ Non-cohab	3397	84.5	621	15.5	2048	94.4	122	5.6	1349	73	499	27
Divorced	1072	83.4	214	16.6	549	94	35	6	523	74.5	179	25.5
Widower	833	82.2	180	17.8	215	96.4	8	3.6	618	78.2	172	21.8
*Sexual orientation*												
Hetero-sexual	18,736	87.4	2700	12.6	9595	96.6	338	3.4	9141	79.5	2362	20.5
LGBQ	562	79.8	142	20.2	266	92.7	21	7.3	296	71	121	29
**Residential context**												
*Municipality size*												
> 50,000	4468	81.8	994	18.2	2352	95	124	5	2116	70.9	870	29.1
10–50,000	6911	85.9	1137	14.1	3610	96.1	147	3.9	3301	76.9	990	23.1
< 10,000	7919	91.8	711	8.2	3899	97.8	88	2.2	4020	86.6	623	13.4
*Residential ownership*												
Resident-owned	14,960	89	1845	11	7720	97.3	215	2.7	7240	81.6	1630	18.4
Rental	3198	81.3	735	18.7	1519	93.3	109	6.7	1679	72.8	626	27.2
Other arrangements	1140	81.3	262	18.7	622	94.7	35	5.3	518	69.5	227	30.5
**Socioeconomic and material conditions**												
*Income quintiles*												
1 lowest	3590	81.1	838	18.9	1466	94.6	84	5.4	2124	73.8	754	26.2
2	3726	84.2	702	15.9	1661	95.4	81	4.7	2065	76.9	621	23.1
3	3889	87.8	539	12.2	1702	96.3	66	3.7	2187	82.2	473	17.8
4	3987	90	441	10	2033	97,3	56	2.7	1954	83.5	385	16.5
5 highest	4106	92.7	322	7.3	2999	97.7	72	2.3	1107	81.6	250	18.4
*Education*												
Low	9099	86.8	1382	13.2	4946	95.9	211	4.1	4153	78	1171	22
Medium	6769	87.4	978	12.6	3603	96.7	122	3.3	3166	78.7	856	21.3
High	3430	87.7	482	12.3	1312	98.1	26	1.9	2118	82.3	456	17.7
*Labour market position*												
Working	9745	89.5	1143	10.5	5005	97.4	133	2.6	4740	82.4	1010	17.6
Studying	1083	76.5	332	23.5	489	94.4	29	5.6	594	66.2	303	33.8
Retired	5219	87.5	745	12.5	2952	96.3	112	3.7	2267	78.2	633	21.8
Unemployed	3251	83.9	622	16.1	1415	94.3	85	5.7	1836	77.4	537	22.6
*Low cash margin*												
No, able to get 15,000 SEK	16,640	88.4	2178	11.6	8756	97.2	253	2.8	7884	80.4	1925	19.6
Yes, not able to get 15,000 SEK	2658	80	664	20	1105	91.3	106	8.8	1553	73.6	558	26.4
*Difficulties to make ends meet*												
No	17,363	88	2360	12	8973	97	279	3	8390	80.1	2081	19.9
Yes	1935	80.1	482	20	888	91.7	80	8.3	1047	72.3	402	27.7
**Psychosocial conditions**												
*Social participation*												
Yes	17,246	87.3	2514	12.7	8602	96.8	288	3.2	8642	79.5	2226	20.5
No	2052	86.2	328	13.8	1257	94.7	71	5.4	795	75.6	257	24.4
*Social trust*												
Yes	15,798	89.2	1914	10.8	7959	97.4	214	2.6	7839	82.2	1700	17.8
No	3500	79	928	21	1902	92.9	145	7.1	1598	67.1	783	32.9
*Subjected to threat or violence*												
No	18,633	87.6	2632	12.4	9550	96.9	307	3.1	9083	79.6	2325	20.4
Yes	665	76	210	24	311	85.7	52	14.3	354	69.1	158	30.9
*Degrading treatment*												
No	16,639	88.9	2069	11.1	8857	97.3	242	2.7	7782	81	1827	19
Yes	2659	77.5	773	22.5	1004	89.6	117	10.4	1655	71.6	656	28.4

C is defined as twice the area between the CC and line of equality. The C is equal to 0 if the CC equals the line of equity (indicating no inequality), but also in cases where the CC crosses the line of equity and the areas below and above the line are cancelling each other out. When CC lies above the line of equity, the value of the C is negative, signifying that the outcome is concentrated amongst the worse off. The value of C is positive when the CC lies below the line of equity, meaning that the outcome is concentrated amongst the better off [30, 31].

To address the second and third aims, decomposition analysis was used [32]. Decomposition of the C is a regression-based analysis where C is decomposed by a set of determinants (see Table 1) [30, 32], and quantifies the individual independent contributions of the included determinants to the C. The C of fear of crime by income was decomposed by all the explanatory factors described above.

The C was decomposed by using the so-called Wagstaff-type of decomposition analysis [30]. Accordingly, the C for any linear additive regression model of health (y), such as:

1 $$ \gamma =\alpha +{\sum}_k{\beta}_k{X}_k+\varepsilon $$

can be expressed as follows:

2 $$ \mathrm{C}={\sum}_k\left({\beta}_k{\overline{X}}_k/\mu \right){C}_k+{GC}_{\varepsilon }/\mu $$

Where μ is the mean or in case of binary factor the proportion of the outcome variable (*y*), *β*_*k*_ is the coefficient for determinants *k* from a linear regression model, ${\overline{X}}_k$is the mean of *X* for *k*, *C*_*k*_  is the concentration index for *X*_*k*_, and *GC*_*ε*_ is the generalised concentration index for the error term (*ε*). As stated in the Eq. (2) ∁ equals the weighted sum of the concentration indices of the k determinants, where the weight for *X*_*k*_ is the elasticity of y with respect to *X*_*k*_ [30] . The last term *GC*_*ε*_/*μ* of the Eq. (2) captures the residual component that expresses the income-related inequalities in the outcome that the systematic variation in the determinants *k* across socioeconomic groups could not explain [30].

As the outcome variable – fear of crime –, was binary, the normalisation procedure suggested by Wagstaff [28, 30, 32] was applied to the decomposition analysis and to the C. The outcome of this present study was binary, thus we applied a statistical technique which was developed for such non-linear settings. The World Bank technical notes proposes using marginal effects from probit models to restore the underlying linear assumptions of the decomposition analysis [30]. Specifically, to substitute the *β*_*k*_ in the Eq. (2) for the marginal effects from a probit model, and thereby use these marginal effects to calculate the contributions of the *k* explanatory variables (determinants *k*) [30]. We chose to apply this method in the present study.

The linear approximation of the non-linear setting can be described as follows:

3 $$ \mathrm{C}={\sum}_k\left({\beta}_k^m{\overline{X}}_k/\mu \right){C}_k+{GC}_{\varepsilon }/\mu $$

The results of the decomposition analysis, summarized as the estimates of coefficient, elasticity, concentration index of the contributing factor, and absolute and relative contributions to the total concentration index and adjusted relative contribution.

The coefficients (*β*_*k*_), are the marginal effects from the probit regression model, show the magnitude of the relationship of the variable with the outcome. The elasticity, depicted as $\left({\beta}_k{\overline{x}}_k/\mu \right)$ in Eq. (2), indicates the frequency weighted marginal effect [30], i.e. the marginal effect multiplied by the mean of the explanatory factor in question (and divided by the mean of the outcome, a constant in the model). Therefore, it might happen that a high (low) coefficient has a low (high) elasticity due to disproportionately low (high) frequency of that variable category. The contributing factors are the determinants of the outcome that theoretically can explain the income inequality of the outcome, the fear of crime variable in this present study. The concentration indices of the contributing factors (CI), denoted as *C*_*k*_ in the Eq (2), are the relative measure of inequality of the contributing factors, thus the same interpretation can be applied here as for the total C, i.e. a negative CI indicates that the category is concentrated among the poor and vice versa. The contribution can be calculated in both absolute and relative terms. The absolute contribution is the multiplicative product of CI (*C*_*k*_) and elasticity $\left({\beta}_k{\overline{x}}_k/\mu \right)$ [30], and is as such expressed on the same scale as the overall concentration index. The relative contributions instead show how much percentage of the inequality in the outcome (C) is attributable to the inequality in the contributing factor. Relative contribution of a factor is calculated by dividing its absolute contribution by the total inequality of C and then multiplying it with 100. Additionally, the adjusted relative contribution expresses the factor contribution in relation to the sum of those contributing in the same direction as the concentration index, i.e. those that contributes towards the observed inequality [30].

Finally, variance inflation factor (VIF) was used to establish whether multicollinearity between the variables was present but all were below the threshold of 5. The dummy retired variable of the labour market position had the highest VIF of 4.71.

All analyses were done on the total sample and stratified by gender to capture any gender-specific patterns (aim 3). All the statistical analyses were performed with Stata 13.0 software.

## Results

Table 1 displays the descriptive statistics on the total and gender stratified sample with absolute and relative frequencies of the studied variables. Fear of crime was reported by *12.8% of the* *total sample: 20.8% among women and 3.5% among men.* In general, there were more individuals who reported fear of crime among those aged 16–29, non-Swedish-born, widower or LBGQ, as well as among those who had rental or other living arrangements, lower income, financial difficulties or were studying. Furthermore, fear of crime was more common among those who reported unfavourable psychosocial conditions or former exposure to threat, violence or degrading treatment, and in those who reported lack of social trust. Overall, the descriptive results thus pointed to fear of crime being more frequent in disadvantaged social groups.

Regarding the first aim, substantial income inequalities were observed among the total population, men and women (C = − 0.219; 95% CIs [− 0.241, − 0.198]; C = − 0.187; 95% CIs [− 0.247, − 0.127]; and C = − 0.132; 95% CIs [− 0.158, − 0.106], respectively). The negative values of these estimates demonstrate that fear of crime was concentrated amongst the worst off, also illustrated by the CCs on total and gender-stratified samples presented in Fig. 2.

**Fig. 3:**
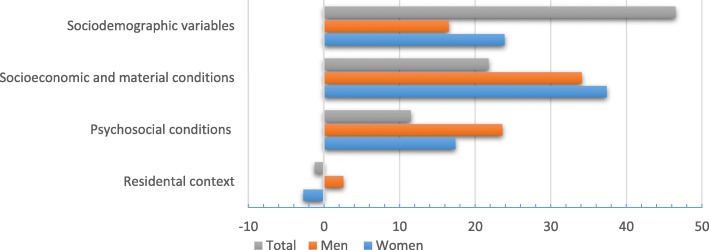
Relative (%) contributions of groups of variables to the concentration index of income-related inequalities in fear of crime in the total sample, men and women in 2014, northern Sweden

Corresponding to the second and third aims, Table 2 and Fig. 3 present the results of the decomposition analysis on the total and gender-stratified samples. Of the overall C, 78.6% (total sample), 76.9% (women) and 76.0% (men) of the inequality was explained by the included variables.

**Table 2:** Summary of decomposition analysis of income-related inequalities in fear of crime in the total sample, men and women in 2014, northern Sweden

N = 22,140	Total					Men					Women				
	Coeff	Elast	CI	Cont to C		Coeff	Elast	CI	Cont to C		Coeff	Elast	CI	Cont to C	
				Abs	Rel				Abs	Rel				Abs	Rel
**Sociodemographic characteristics**															
*Gender (REF: Men)*	0.161***	0.675	−0.118	−0.079	36.2										
*Age (REF:45-64 yrs)*															
16–29 yrs	0.024**	0.028	−0.504	−0.014	6.5	−0.013**	−0.051	−0.504	0.026	−13.8	0.076***	0.061	−0.498	−0.030	23.1
30–44 yrs	0.011	0.018	0.192	0.003	−1.6	−0.006	−0.037	0.209	−0.008	4.1	0.032**	0.035	0.200	0.007	−5.3
65–84 yrs	0.023**	0.058	−0.141	− 0.008	3.7	0.009	0.091	−0.135	−0.012	6.6	0.040*	0.059	−0.183	−0.011	8.1
*Country of birth (REF: Sweden)*	0.011	0.005	−0.171	− 0.001	0.4	0.015*	0.021	−0.208	−0.004	2.3	0.003	0.001	−0.121	0.000	0.1
*Civil status (REF: Married/cohab)*															
Unmarried/not cohab	0.004	0.005	−0.311	−0.002	0.7	0.013**	0.084	−0.359	−0.029	15.4	−0.013	−0.010	−0.295	0.003	−2.2
Divorced	0.023**	0.010	−0.002	0.000	0.0	0.009	0.015	−0.073	−0.001	0.6	0.040*	0.011	0.070	0.001	−0.6
Widower	0.007	0.003	−0.174	0.000	0.2	0.001	0.001	−0.198	0.000	0.1	0.018	0.006	−0.093	−0.001	0.4
*Sexual orientation (REF: Heterosexual)*	0.008	0.002	−0.328	−0.001	0.3	0.009	0.007	−0.346	−0.002	1.3	0.004	0.001	−0.311	0.000	0.2
**Subtotal**				**−0.102**	**46.5**				**−0.031**	**16.5**				**−0.031**	**23.9**
**Residential context**															
*Municipality size (REF: < 10,000)*															
10,000–50,000	0.063***	0.179	0.026	0.005	−2.1	0.018***	0.193	0.028	0.005	−2.9	0.112***	0.194	0.022	0.004	−3.3
> 50,000	0.108***	0.208	0.046	0.010	−4.4	0.031***	0.216	0.056	0.012	−6.5	0.184***	0.221	0.046	0.010	−7.7
*Residential ownership (REF: Resident-owned)*															
Rental	0.024**	0.034	−0.195	−0.007	3.0	0.016***	0.073	−0.216	−0.016	8.5	0.034**	0.031	−0.168	−0.005	4.0
Other arrangements	0.019*	0.009	−0.525	−0.005	2.2	0.007	0.012	−0.549	−0.007	3.6	0.036*	0.011	−0.514	−0.006	4.2
**Subtotal**				**0.003**	**−1.2**				**−0.005**	**2.6**				**0.004**	**−2.7**
**Socioeconomic and material conditions**															
*Income quintiles (REF: Highest)*															
4	−0.006	−0.009	0.400	−0.004	1.6	0.000	0.003	0.195	0.000	−0.3	−0.019	−0.018	0.576	−0.010	7.7
3	−0.006	−0.008	0.000	0.000	0.0	0.002	0.009	−0.183	−0.002	0.9	−0.022	−0.023	0.157	−0.004	2.8
2	0.008	0.012	−0.400	−0.005	2.1	0.000	−0.002	−0.526	0.001	−0.6	0.005	0.005	−0.292	−0.001	1.1
1	0.007	0.011	−0.800	−0.009	4.2	0.000	0.001	−0.848	0.000	0.2	0.004	0.004	−0.759	−0.003	2.5
*Education (REF: High)*															
Medium	0.009	0.026	0.062	0.002	−0.7	0.015*	0.152	0.070	0.011	−5.7	0.011	0.018	0.047	0.001	−0.7
Low	0.017**	0.061	−0.148	− 0.009	4.1	0.017**	0.250	−0.126	−0.031	16.8	0.022*	0.047	−0.197	−0.009	7.1
*Labour market position (REF: Working)*															
Studying	0.030**	0.015	−0.671	− 0.009	4.5	0.007	0.010	−0.755	−0.007	4.0	0.058***	0.021	−0.597	−0.013	9.6
Retired	0.012	0.025	−0.156	−0.004	1.8	0.003	0.028	−0.163	−0.005	2.5	0.022	0.026	−0.195	−0.005	3.8
Unemployed	0.008	0.011	−0.276	−0.003	1.4	0.004	0.016	−0.297	−0.005	2.6	0.011	0.011	−0.243	−0.003	2.0
*Low* *cash margin (REF: No, able to get 15K*^*1*^)	0.006	0.007	−0.339	−0.003	1.1	0.016**	0.054	−0.411	−0.022	11.8	−0.003	−0.003	−0.265	0.001	−0.5
*Difficulties to make ends meet (REF: No)*	0.016*	0.014	−0.240	−0.003	1.5	0.004	0.012	−0.309	−0.004	2.0	0.027*	0.016	−0.169	−0.003	2.0
**Subtotal**				**−0.047**	**21.8**				**−0.064**	**34.1**				**−0.049**	**37.4**
**Psychosocial conditions**															
*Social participation (REF: Yes)*	0.013	0.011	−0.225	− 0.002	1.1	0.003	0.012	−0.254	−0.003	1.7	0.018	0.008	−0.255	−0.002	1.5
*Social trust (REF: Yes)*	0.063***	0.098	−0.169	−0.017	7.6	0.020***	0.116	−0.160	−0.019	9.9	0.110***	0.105	−0.187	−0.020	14.9
*Subjected to threat or violence (REF: No)*	0.050***	0.015	−0.081	−0.001	0.6	0.052***	0.053	−0.168	−0.009	4.7	0.042*	0.009	0.011	0.000	−0.1
*Degrading treatment (REF: No)*	0.050***	0.060	−0.083	−0.005	2.3	0.043***	0.136	−0.100	−0.014	7.2	0.062***	0.057	−0.023	−0.001	1.0
**Subtotal**				**−0.025**	**11.5**				**−0.044**	**23.6**				**−0.023**	**17.4**
**Inequality (total)**				**−0.219**	**100**				**−0.187**	**100**				**−0.132**	**100**
**Standard error**				**0.011**	**5.0**				**0.030**	**16.2**				**0.013**	**10.0**
**Residual**				**−0.047**	**21.6**				**−0.043**	**23.1**				**−0.032**	**24.0**
**Inequality (explained)**				**−0.172**	**78.4**				**−0.144**	**76.9**				**−0.100**	**76.0**

*Coeff* Marginal effects from the probit model, *Elast* elasticity, *CI* Concentration index of the determinants, *Cont to C* Contribution to the total inequality, *Abs* Absolute contribution, *Rel* Relative contribution calculated on the overall explained proportion of total inequality

^1^15,000 Swedish Krona (SEK) equals to ≈2172 US$, based on exchange rate from January 1, 2012

* indicates <*p* 0.05; ** indicates *p* < 0.01; *** indicates *p* < 0.001

In the total sample, the sociodemographic characteristics together contributed the most, amounting to 46.5% of the income inequality in fear of crime, while in the stratified samples the same group of explanatory factors but excluding gender, contributed with 23.9 and 16.5% for women and men, respectively. Such a high contribution in the total sample was mainly attributed to the individual contribution of 36.2% by gender, and a less sizable contribution came from age with 8.6%. For women, age independently contributed positively with 25.9%, which was the highest contributing factor in this group of variables. In contrast, in men age counteracted the inequalities, so its net contribution to the inequality was insubstantial. Instead, the most important contributing factor for men was the unmarried/not cohabitating variable with 16.1%.

The contribution of socio-economic and material conditions was the second most important set of variables in the total sample, with a 21.8% contribution to the inequality, of which the individual contributions of the specific variables were rather small. In the stratified sample, this group of factors was instead the most dominant one, contributing with 37.4% in women and 34.1% among men. In women, the strongest contributing factors were education, labour market position and income quintiles, and in men, low cash margin also displayed a large contribution in addition to education and labour market position,

The psychosocial factors accounted for 11.5% in the total sample. Contrary to the previous two instances, psychosocial factors were numerically more important for the inequalities in men (23.6%) than in women (17.4%). For all three samples, social trust was the major contributing variable: 7.6% in total sample, 9.9% in men, and 14.9% in women. Degrading treatment was a quite important factor for men, explaining 7.2% of the total inequality, while it had a marginal contribution for the total sample and women. The residential context was the least vital (− 2.7% in women and 2.6% in men).

## Discussion

To the authors’ knowledge, this is the first population-based study aimed at exploring income inequalities in fear of crime and the determinants of these inequalities. The results indicate substantial income inequalities in fear of crime to the disadvantage of the less well-off, and that these inequalities were largely attributable to a range of social determinants, with partly different patterns in women and men. In our study crime was limited to ‘fear of being assaulted, robbed or otherwise victimized’, but there are several other types of crime e.g.: violent burglary, gang violence, arson, cybercrime, domestic abuse etc. which did not fall under our scope. Therefore, we limit our findings only to fear of crime as it was defined in our conceptual framework.

Our finding of income inequality in fear of crime reflects that fear of crime is more common among those who are socially disadvantaged, which corresponds with the literature [3–5], and adds to the observation of Vieno et al. [5] that income equality in a society is associated with low levels of fear of crime. However, we also found that the best-off women experienced slightly greater fear of crime than did the middle-income women. A possible reason for this could be that those who are best-off have the most to lose in terms of property in an incidental victimization, and in such situation women are viewed as more vulnerable compared to men.

A pervasive finding throughout the analyses was indeed that fear of crime was highly gendered, with the prevalence of fear of crime among women almost six times higher than in men, but with a slightly larger income inequality in men than in women. Moreover, the gender-stratified analyses also showed notable differences in the degree of income inequality between the total population, men and women, where the total population had the highest inequality – most probably due to a reflection of gender explaining a large portion of the inequality, since women had lower income and also reported a higher level of fear of crime – while men reported a higher inequality than women. Taken together, the finding emphasizes the intertwinement of gender and (inequalities in) fear of crime, as suggested by the literature [10, 18] and by our conceptual model in Fig. 1. As noted in the introduction, such an ubiquitous role of gender may be rooted in hegemonic ideologies of femininity and masculinity [10].

Sociodemographic characteristics, exemplifying the position in the social space in Fig. 1, seemed to be the most important group of factors explaining inequalities in the total population, and as noted above, gender was a particularly dominant contributor. The literature has also established a prominent role of age and income among the determinants of inequalities in fear of crime [4–6, 33, 34]. Our findings suggest that socio-economic and material conditions contributed considerably to the inequalities, particularly in the gender-stratified analysis, where education, low cash margin in men and labour market position in women emerged as strong contributors. Recent research from the European region [6] suggests that younger women and the elderly – groups which also tend to have lower income – have a higher proportion of fear of crime, findings that resonate with the mentioned female oppression and perceived vulnerability theory [10, 12]. In accordance with this, our study found that the age groups 16–29 in women and 65–84 in both genders are important factors in the explanation of income inequalities in fear of crime. Corresponding with vulnerability theory [34, 35], these groups might feel themselves unprotected against an eventual crime through belonging to the less affluent part of society, which could provide an explanation to their contribution to income inequality in fear of crime.

Psychosocial conditions seemed to have a moderate importance in explaining income inequalities in fear of crime, with social trust and degrading treatment – already established as important determinants for fear of crime [16, 36] – being prominent contributors, in particular. As depicted in Fig. 1, psychosocial conditions, such as degrading treatment, might make individuals more susceptible to fear of crime, which in turn may contribute to negative health outcomes, for example depression or anxiety. Residential context emerged as the least important group of explanatory factors in this present study. Although residential environment can be an important upstream risk factor for fear of crime [14, 15, 18, 37], it might not be independently relevant to reducing the income inequalities in fear of crime, taking into account the other factors in the model.

According to the Gender Gap Report 2016 [38] Sweden was ranked fourth by closing more than 81% of its gender gap. However, such measures do not take into account victimization and fear as a gendered phenomenon, giving a false impression of an equitable setting when it comes to gender. The present study instead paints a worrisome picture where prevalence of and income inequality in fear of crime are both substantial and highly gendered in the northern Swedish setting. Our findings imply a need for a strengthened gender and public health perspective on inequalities in fear of crime, to provide a safe life for all.

### Methodological considerations

Strengths of the study included the large population-based sample with linked register data, which might decrease potential reporting bias. Selection bias might also be an issue since the response rate was around 50%. Moreover, we cannot disregard that the outcome, fear of crime, might be underreported [39].

While the selection of the explanatory variables was in accordance with the conceptual framework depicting a hypothetical causal chain (Fig. 1), the cross-sectional design and analytical methods do not allow for causal inference, which is the ever-present drawback of cross-sectional studies. The possibility of feedback loops or unconsidered third variables is still unaccounted for, and presents a challenge for any empirical research investigating complex phenomena. The decomposition analysis do not support stronger causal inference than linear regression, and should therefore should be seen as descriptive rather than causal.

The secondary data was not specifically collected for this study. Therefore, not all the plausible determinants could be included, which reflects the 21% unexplained residual of the inequalities in the total population. Moreover, although the outcome variable fear of crime has been used by the Public Health Agency of Sweden for monitoring purposes since 2005, has been used in previous research [24, 36, 40], and is similar to other measures used in the literature [4, 13, 15, 41], it has not been formally validated, which could introduce error in the estimates. Additionally, a conceptual framework is at best an approximate and simplified depiction of a complex reality, and the one used as point of departure for the present study might have led to the exclusion of unconsidered determinants of income inequality in fear of crime.

## Conclusions

The present study shows that income inequality in fear of crime exists even in a comparatively equitable setting like northern Sweden. One’s position in social space involves a risk for a range of socio-economic and psychosocial exposures that are directly linked to fear of crime, where gender inequity seems to be the most central aspect for fear of crime and its income-related inequality. The existing gender inequity needs to be treated as a greater structural level problem together with socio-economic inequalities, to mitigate fear of crime and thereby potentially inequalities in health too. In order to reduce the income inequalities in fear of crime for both genders, policymakers should prioritize intervening at the structural level, for example by empowering women from all socio-economic backgrounds and ensuring a safe public space for all, as supported by actual legislation.

## Abbreviations

C/Cs: Concentration index(es)
CC/CCs: Concentration curve(s)
CIs: Confidence intervals
HET: Health on Equal Terms
LGBQ: Lesbian-gay-bisexual-questioning
SEK: Swedish Krona
VIF: Variance inflation factor
